# Co-occurrence of dental caries and periodontitis: multilevel modelling approach

**DOI:** 10.1186/s12903-024-03918-2

**Published:** 2024-01-31

**Authors:** Lina Stangvaltaite-Mouhat, Rasa Skudutyte-Rysstad, Hayley Ko, Indre Stankeviciene, Jolanta Aleksejuniene, Alina Puriene

**Affiliations:** 1 Oral Health Centre of Expertise in Eastern Norway, Sørkedalsveien 10A, Oslo, 0369 Norway; 2 Institute of Dentistry, Faculty of Medicine, Vilnius University, M. K. Čiurlionio 21, Vilnius, 03101 Lithuania; 3 Department of Preventive and Community Dentistry, Faculty of Dentistry, The University of British Columbia, Vancouver, Canada

**Keywords:** Dental caries, Adults, Periodontitis, Association, Tooth, Multilevel modelling

## Abstract

**Background:**

Previous studies reported varyingly positive, negative, or no relationships between caries and periodontitis. Therefore, the aim was to assess the potential co-occurrence of caries experience and periodontal inflammation on the same teeth.

**Methods:**

This cross-sectional study used data from the Lithuanian National Oral Health Survey. The study included a stratified random sample of 1405 individuals aged 34–78, recruited from 5 Lithuanian cities and 10 peri-urban/rural areas (response rate 52%). Information about sociodemographic (age, sex, education, residence), behavioral (sugar-containing diet, tooth brushing frequency, use of interdental care products, last dental visit, smoking) and biological (systemic disease, use of medication and xerostomia) determinants was collected using the World Health Organization (WHO) Oral Health Questionnaire for Adults supplemented with additional questions. Clinical data were recorded using the WHO criteria and collected by one trained and calibrated examiner. Dental caries status was recorded as sound, decayed, missing, filled surfaces. Subsequently for the analyses, status was recorded at a tooth-level as decayed- and filled-teeth (DT and FT) including proximal, buccal, and oral surfaces. Two measures were used for periodontal status. The probing pocket depth (PPD) was measured at six sites and recorded at a tooth level into the absence of PPD or presence of PPD ≥ 4 mm. Bleeding on probing (BOP) was measured at the same six sites and was recorded as either present or absent at a tooth-level. Univariable and multivariable 2-level random intercept binary logistic regression analyses were utilized.

**Results:**

Positive associations were found between DT and BOP (OR 1.42, 95% CI 1.20–1.67), FT and BOP (OR 2.07, 95% CI 1.82–2.23), DT and PPD (OR 1.38, 95% CI 1.15–1.67) and FT and PPD (OR 2.01, 95% CI 1.83–2.20).

**Conclusions:**

Our findings add evidence for the co-occurrence of periodontal inflammation and caries on the same teeth. This suggests the need for increased emphasis on a transdisciplinary approach in designing oral health interventions that target dental caries and periodontal disease simultaneously. In addition, longitudinal studies exploring the co-occurrence of caries and periodontal disease at the same sites, taking into consideration the levels of both conditions and genetic variation, are warranted.

**Supplementary Information:**

The online version contains supplementary material available at 10.1186/s12903-024-03918-2.

## Background

According to the Global Burden of Diseases 2017 report, dental caries and periodontitis are among the most prevalent non-communicable conditions [1]. Despite being preventable, these conditions are the primary causes of tooth loss [2], leading to compromised nutrition [3], psychosocial disability, lower self-esteem [4], and a generally impaired quality of life [5].

According to the World Health Organization’s common risk factor approach, non-communicable conditions share the same social and behavioral determinants and thus might occur simultaneously in the same patients. For caries and periodontitis, known common determinants are oral biofilms, an unhealthy lifestyle, compromised general health, and a lower socioeconomic position [6]. The common risk factor approach was supported by the findings of two cross-sectional Finnish adult studies indicating common risk factors for caries and periodontitis, such as oral biofilms, smoking, lack of regular dental visits, older age and lower education [7]. Recent studies focused on Canadian, Chilean, Chinese and Spanish adults also demonstrated the co-occurrence of periodontal disease and dental caries in the same individuals [8–11].

Earlier studies suggested a positive relationship between compromised restorations and gingival bleeding or increased pocket probing depth [12–14]. In a longitudinal adolescent study, restorations and untreated carious lesions were associated with gingival inflammation and decreased alveolar bone height [15]. The prevailing explanation suggested that changes in biofilm composition due to a lesion or a restoration lead to periodontal disease.

On the other hand, no association between periodontal disease and caries was found in German and Scottish adults [16, 17]. A negative association between juvenile periodontitis and untreated carious lesions on proximal surfaces has also been reported [18, 19], explained by the presence of antagonistic oral biofilms in dental caries and juvenile periodontitis. Another explanation for the juvenile periodontitis was attributed to the potential genetic effect of ethnicity [20].

A joint meeting of the European Federation of Periodontology and the European Association for Caries Research reviewed the existing evidence for caries and periodontal diseases and called for more research to explore their co-occurrence [6, 21]. Therefore, the aim of this study was to examine the co-occurrence of caries and periodontitis at the same teeth. The null hypothesis indicated no association between dental caries (measured by decayed teeth and filled teeth) and periodontal disease (measured by bleeding on probing and pocket probing depth).

## Methods

### Study design and participants

This cross-sectional study analyzed data from the Lithuanian National Oral Health Survey 2017/2019, which included a random stratified sample of 1405 individuals aged 34–78. Participants were recruited from the five biggest Lithuanian cities and ten randomly selected peri-urban/rural areas, one from each of the ten Lithuanian counties (response rate 52%) [22, 23].

### Questionnaire

The information about the sociodemographic, behavioral and biological determinants was collected using the World Health Organization (WHO) Oral Health Questionnaire for Adults [24] which was supplemented with questions regarding the presence of systemic diseases, use of medications, and symptoms of xerostomia. Sociodemographic determinants included age, sex, years of education, and residence (urban vs. rural/peri-urban). Behavioral determinants encompassed the frequency of sugar-containing diet, tooth brushing frequency (twice a day or more vs. once a day or less), use interdental care products (interdental brush and/or floss vs. no), the last dental visit (within the last year vs. more than a year ago), and smoking habits (daily vs. not daily). The sugar-containing diet was calculated as follows: Participants were asked to indicate the frequency of consuming eight different groups of sugar-containing products on a 6-point scale, where 1 meant ‘rarely/never’, 2 ‘several times a month’, 3 ‘once a week’, 4 ‘several times a week’, 5 ‘every day’, and 6 ‘several times a day’. The corresponding frequency values were added and grouped into ‘less frequent’ and ‘more frequent’ using the median value of 20 as a cut-off point. Individual level biological determinants were as follows: self-reported presence of systemic disease (yes vs. no), regular use of medications (yes vs. no) and xerostomia (never/seldom vs. often/always).

### Clinical measurements

The dental examination adhered to the WHO Oral Health Survey Basic Methods recommendations. It was conducted in dental offices using a dental chair, a dental unit light, a CPITN periodontal probe, and an oral mirror. For all participants, one trained and calibrated examiner (IS) performed all clinical examinations on all teeth excluding third molars. Dental caries status was recorded as sound, decayed, missing, or filled surfaces, following the WHO criteria, i.e., clinically visible caries was registered only at the cavitation level [24]. In the present study, caries was registered on proximal, buccal, and oral surfaces. For statistical analyses, caries was recoded into decayed teeth (DT) and filled teeth (FT). Two periodontal measures were used. Pocket probing depth (PPD) was measured at six sites (disto-buccal, buccal, mesio-buccal, mesio-oral, oral and disto-oral) and recorded at a tooth level measurement indicating an absence of condition, PPD 4–5 mm, or PPD ≥ 6 mm. For the analyses, data was dichotomized into absence of condition vs. presence of condition (≥ PPD 4 mm). Bleeding on probing (BOP) was measured at the same six sites and recorded as presence or absence of the condition at a tooth level.

The intra-examiner agreement was assessed at a surface level based on duplicate recordings of 10 individuals. The intra-class correlation values were as follows: 100% for DS, 100% for FS, 94% for PPD 4 mm and 100% for PPD 6 mm.

### Statistics

The SPSS version 27 (IBM, Armonk, NY, USA) was used for statistical analyses. Means and standard deviations (SD) were calculated for continuous variables and frequencies for categorical tooth-level and individual-level variables. Two-level random intercept binary logistic regression analyses were used to assess the association between the outcomes (PPD and BOP) and the variables (DT and FT), concomitantly accounting for the inherent clustering of the data, where teeth information (Level-1) was nested within individuals (Level-2). DT and FT were Level-1 (tooth-level) variables, whereas individual-level variables were Level-2 variables.

Model 0, an “empty” model, was used to ascertain the presence of the clustering effect. Model 1, a two-level model, allowed for the clustering of teeth data within individual data and included one of the tooth-level variables at a time, i.e., DT or FT. Model 2 included individual-level variables, which are shown in a directed acyclic graph (Supplementary Fig. 1). Since sociodemographic variables, education, and residency were considered distal determinants for caries and periodontitis with their effect manifesting at least partly through oral health behaviors, education and residency were not adjusted for in the multivariable analyses. Observations with missing data were excluded. A sensitivity analysis, including all covariates, was performed and did not substantially affect the estimates of association between caries and periodontitis parameters (Supplementary Table 1).

In Model 2, age stratified (≤ 44, 45–54, 55–64, 65 + age groups) subgroup analyses were also performed.

The thresholds for statistical significance for all analyses were set at *p* < 0.05, and odds ratios (OR) are presented with their 95% confidence intervals (CI). The model fit was calculated and assessed by the Bayesian Information Criterion (BIC) and the interclass correlation coefficient (ICC), which describes how much of the variance in the outcome was attributable to within-individual variation, was calculated for each of the models.

## Results

### Characteristics of participants and tooth-related variables

Data were available for 1405 participants, encompassing a total of 29,667 teeth. The mean age of participants was 54.9 years (SD 11.9), and the majority were women and urban residents (Table 1). Caries experience and periodontal inflammation were highly prevalent in the study sample; almost half (46%) of the sample’s teeth were decayed or filled, two-thirds (63%) exhibited BOP, and every third tooth (28%) had PPD ≥ 4 mm (Table 2).

**Table 1:** Distribution of participants in relation to sociodemographic, behavioral and biological individual-level variables stratified by age

Individual-level variables (level-2)	≤ 44 years n = 356		45–54 years n = 312	55–64 years n = 362		65 + years n = 375	
Age, years [mean (SD)]	39.03 (2.92)	49.96 (2.85)			59.44 (2.87)		69.54 (3.01)
Sex [n (%)]	356	311			362		375
*Females*	226 (64)	207 (66)			250 (69)		256 (68)
*Males*	130 (36)	104 (34)			112 (31)		119 (32)
Education, years [mean (SD)]	16.52 (3.61)	14.39 (2.87)			14.19 (2.56)		13.87 (3.11)
Residency [n (%)]	356	312			362		374
*Urban*	289 (81)	208 (67)			234 (65)		274 (73)
*Peri-urban/rural*	67 (19)	104 (33)			128 (35)		100 (27)
Sugar-containing diet [n (%)]	299	233			230		204
*Less frequent*	148 (49)	93 (40)			92 (40)		74 (36)
*More frequent*	151 (51)	140 (60)			138 (60)		130 (64)
Tooth brushing frequency [n (%)]	353	309			356		370
*Twice a day or more*	189 (53)	157 (51)			169 (47)		172 (46)
*Once a day or less*	164 (47)	152 (49)			187 (53)		198 (54)
Use of interdental care products [n (%)]	352	308			353		368
*Yes*	102 (29)	111 (36)			158 (45)		152 (41)
*No*	250 (71)	197 (64)			195 (55)		216 (59)
Last dental visit [n (%)]	353	309			356		364
*Within the last year*	267 (75)	223 (72)			257 (72)		240 (66)
*More than a year ago*	88 (25)	86 (28)			99 (28)		124 (34)
Smoking [n (%)]	352	304			353		346
*Not daily*	288 (82)	251 (83)			302 (86)		329 (95)
*Daily*	64 (18)	53 (17)			51 (14)		17 (5)
Systemic diseases [n (%)]	341	296			352		346
*No*	238 (70)	153 (52)			226 (64)		74 (21)
*Yes*	103 (10)	143 (48)			126 (36)		272 (79)
Use of medications [n (%)]	348	302			349		352
*No*	286 (82)	183 (61)			139 (40)		77 (22)
*Yes*	62 (18)	119 (38)			210 (60)		275 (78)
Xerostomia [n (%)]	356	312			362		375
*Never/seldom*	345 (97)	293 (94)			328 (90)		327 (87)
*Often/always*	11 (3)	19 (6)			34 (10)		48 (13)

**Table 2:** Distribution of tooth-level variables stratified by age

Tooth-level variables (Level-1)	Total	≤ 44 years	45–54 years	55–64 years	65 + years
	n = 29,667	n = 9168	n = 7309	n = 7187	n = 6003
Periodontal inflammation parameters	n (%)				
Bleeding on probing (BOP)					
*Absence of condition*	10,916 (37)	3470 (38)	2844 (39)	2563 (36)	2039 (34)
*Presence of condition*	18,751 (63)	5698 (62)	4465 (61)	4624 (64)	3964 (66)
Probing pocket depth (PPD) ≥ 4 mm					
*Absence of condition*	21,430 (72)	7682 (84)	5303 (73)	4695 (65)	2795 (47)
*Presence of condition*	8237 (28)	1486 (16)	2006 (27)	2492 (35)	3208 (53)
Dental caries experience	n (%)				
*Sound teeth*	16,139 (54)	5895 (64)	3913 (54)	3536 (49)	2795 (47)
*Decayed and filled teeth (DFT)*	13,528 (46)	3273 (36)	3396 (46)	3651 (51)	3208 (53)
*Filled teeth (FT)*	11,850 (42)	2849 (33)	2985 (43)	3212 (48)	2804 (50)
*Decayed teeth (DT)*	1678 (9)	424 (7)	411 (9)	439 (11)	404 (13)

### Variability of periodontal inflammation

When BOP was used as a measure of the periodontal inflammation, the individual-based ICC was 0.28, indicating that 28% of the variance in BOP was attributable to variation among individuals and 72% within individuals. When PPD was used as an outcome, the ICC estimate among individuals amounted to 0.48 and within individuals to 0.52.

### Co-occurrence of carious lesions and periodontal inflammation on the same teeth

**Fig. 1:**
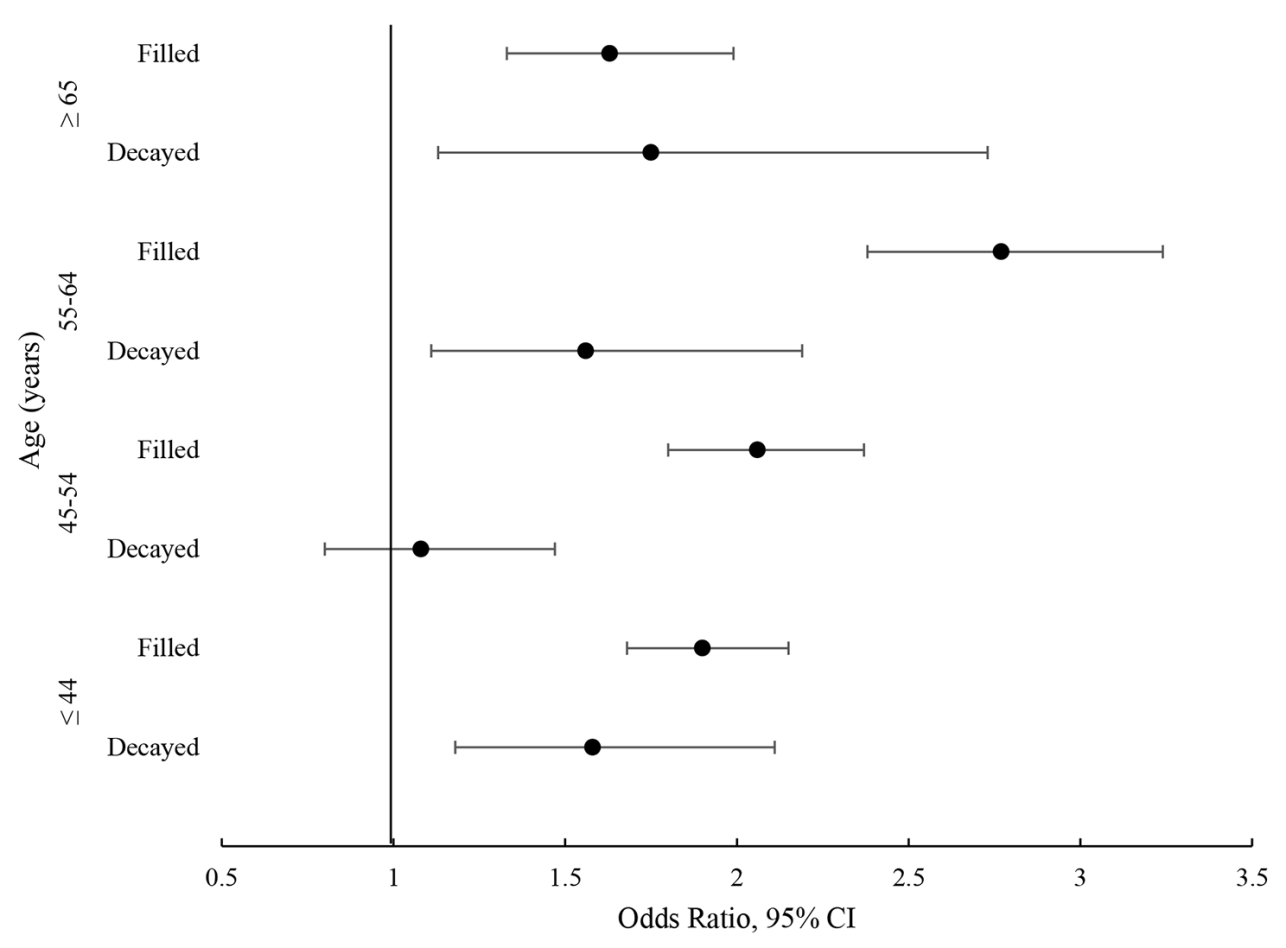
Forest plot of the association between decayed- (DT) and filled teeth (FT) and bleeding on probing (BOP) stratified by age (≤ 44 years, 45–54 years, 55–64 years, ≥ 65 years). Odds ratios with their 95% confidence intervals (CI) for BOP from the two-level random intercept models adjusted for age, sex, sugar-containing diet, toothbrushing frequency, use of interdental care products, last dental visit, smoking, systemic diseases, use of medications, and xerostomia

**Fig. 2:**
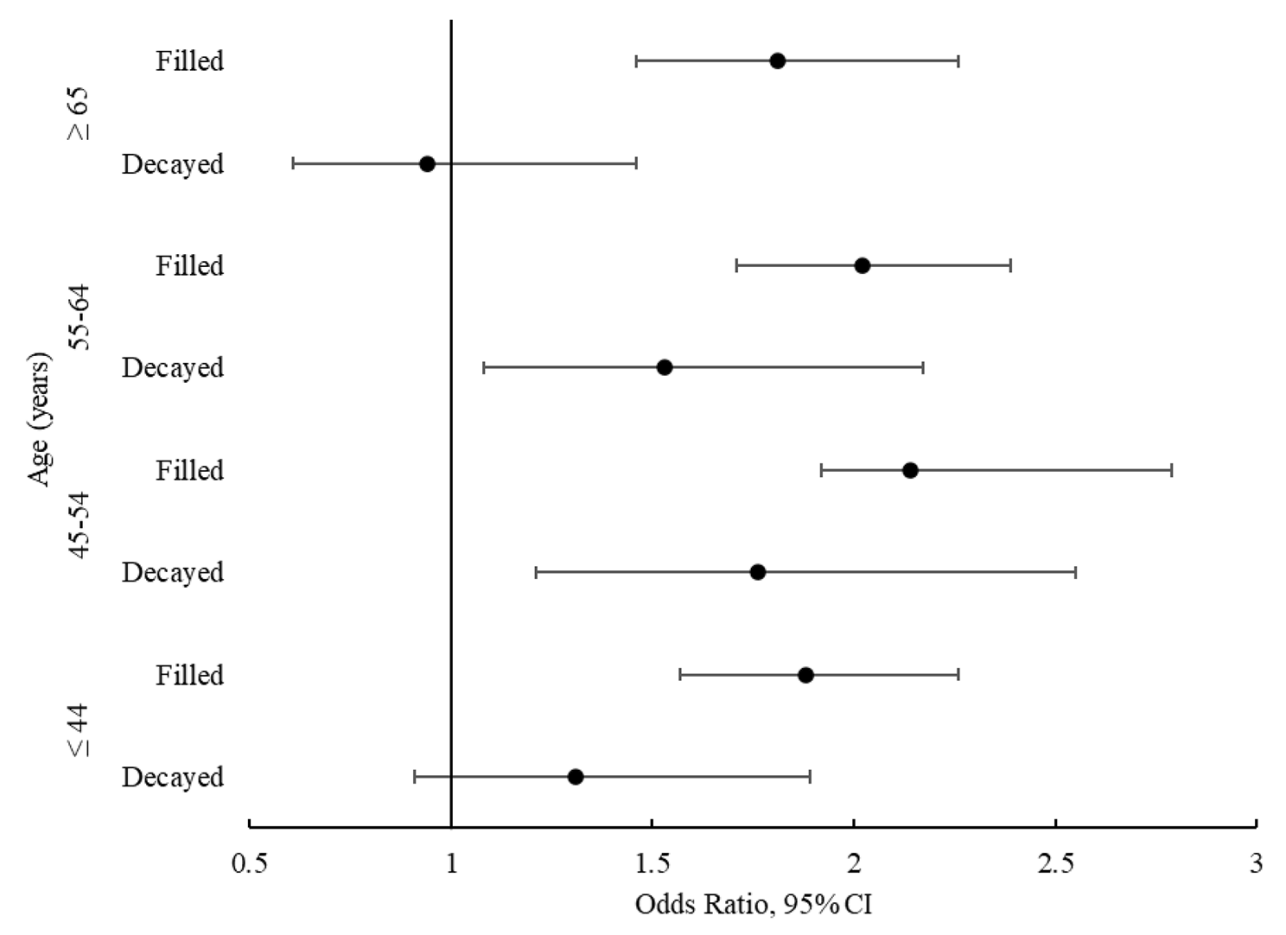
Forest plot of the association between decayed teeth (DT) and filled teeth (FT) and pocket probing depth (PPD) stratified by age (≤ 44 years, 45–54 years, 55–64 years, ≥ 65 years). Odds ratios with their 95% confidence intervals (CI) for BOP from the two-level random intercept models adjusted for age, sex, sugar-containing diet, toothbrushing frequency, use of interdental care products, last dental visit, smoking, systemic disease, use of medication, and xerostomia

According to the multivariable, multilevel binary logistic regression (Model 2), there was a co-occurrence of DT and BOP (OR 1.42, 95% CI 1.20–1.67) and of DT and PPD (OR 1.38, 95% CI 1.15–1.67) on the same teeth (Table 3). The presence of DT increased odds for BOP by 42% and for PPD by 38%. Subgroup analyses demonstrated that the positive association between DT and BOP remained significant in all age groups except for those aged 45–54 years, while the association between DT and PPD was significant only in the age groups 45–54 years and 55–64 years (Figs. 1 and 2).

**Table 3:** Odds ratios (OR) and their 95% Confidence Intervals (CI) for the association between dental caries experience (decayed teeth (DT) and filled teeth (FT)) and periodontal inflammation (bleeding on probing (BOP) and pocket probing depth (PPD)) according to the two-level random intercept binary logistic regression analyses

	Zero model (0)	Main effect (1) only tooth level	Main effects (2) tooth-level and individual-level^i^
***Outcome BOP***			
**Fixed effects**	**OR (95% CI)**		
*No DT (ref.)*			
*DT*		1.62 (1.41–1.85)	1.42 (1.20–1.67)
ICC	0.2815	0.2863	0.2681
Model fit BIC	133950.147	79931.581	55706.451
**Fixed effects**	**OR (95% CI)**		
*No FT (ref.)*			
*FT*		2.02 (1.90–2.14)	2.07 (1.82–2.23)
ICC	0.2815	0.2854	0.2610
Model fit BIC	133950.147	126821.384	87253.791
***Outcome PPD***			
**Fixed effects**	**OR (95% CI)**		
*No DT (ref.)*			
*DT*		1.52 (1.31–1.76)	1.38 (1.15–1.67)
ICC	0.4752	0.4805	0.4176
Model fit BIC	146459.355	88802.983	63309.641
**Fixed effects**	***OR*** **(95% CI)**		
*No FT (ref.)*			
*FT*		2.04 (1.90–2.20)	2.01 (1.83–2.20)
ICC	0.4752	0.4873	0.4113
Model fit BIC	146459.355	139539.056	97715.054

ICC, interclass correlation coefficient; BIC, Bayesian Information Criterion; *ref*., reference category

^i^Analyses adjusted for age, sex, sugar-containing diet, tooth brushing frequency, use of interdental care products, last dental visit, smoking, systemic disease, use of medication and xerostomia

### Co-occurrence of restorations and periodontal inflammation on the same teeth

The positive association (co-occurrence) was also observed between FT and BOP (OR 2.07, 95% CI 1.82–2.23) (Table 3), the presence of FT increased odds for BOP by 81%, and the association was gradually stronger in older age groups except for the oldest age group (Fig. 1). The association between FT and PPD was positive (OR 2.01, 95% CI 1.83–2.20), the presence of FT doubled odds for PPD, and this association remained significant in all age groups (Table 3; Fig. 2).

## Discussion

The present study demonstrated the co-occurrence of caries and periodontal inflammation on the same teeth in a nationally representative sample of 34-78-year-olds. Therefore, the null-hypothesis was rejected. The co-occurrence was more pronounced for treatment experience (FT) than for untreated disease (DT), and this persisted across all age groups. The findings of the current study add to the evidence for the joint manifestation of caries and periodontitis on the same teeth in a population with high levels of both oral diseases.

The results of our study are in line with a previous longitudinal 3-year follow-up adolescent study that also employed multilevel modelling and demonstrated that untreated carious lesions and defective dental restorations were associated with gingival inflammation. The study also found that untreated carious lesions and restorations (both defective and non-defective) were associated with the progression of bone loss [15]. In our study, the associations between periodontal inflammation were stronger with the filled than decayed teeth – a pattern that was not observed in the study in adolescents. Additionally, Albandar and colleagues noted that associations with gingival inflammation were stronger compared to the associations with alveolar bone height – a trend that was not observed in our study. This may be explained by age-related differences in disease levels in the populations studied. It is important to take into consideration that the levels of untreated carious lesions in the aforementioned adolescent population were almost identical to levels observed in our adult population (around 9% and 9%, respectively), however the levels of restorations were much lower (around 10% versus 42%), while the information on the periodontal status was limited in the Albandar et al. adolescent study [15]. Even though dental restorative materials have improved during the last decades, presence of restorations favours biofilm accumulation [25]. Moreover, the biofilm accumulation and its maturation are greatly influenced by the composition of restorative materials, surface properties and roughness [26–28]. Our study was not able to explore specific reasons for association between restorations and periodontal inflammation, as details regarding the restoration material and the quality of restorations were not recorded. In Lithuania, the prevailing restorative material is resin-based composite and previous Lithuanian study in adolescents demonstrated 11% of restorations having not acceptable surface quality [29]. Secondary carious lesions, which can be causally associated with defective restorations [30], were classified as carious lesions in our study. Therefore, at least indirectly, the potential defectiveness of the restorations was accounted for in our study, however exploring mechanisms for observed associations was partly constrained by the lack of more detailed data. The accumulation of biofilm, whether due to defective, non-defective, or unrestored cavities, may be a significant risk factor for periodontal diseases.

The present study demonstrated that 28% of variance in bleeding on probing and 48% of the variance in periodontal pockets were attributable to between-individual variation, respectively. The majority of the explained variance in marginal gingival inflammation (72%) and in the presence of periodontal pockets (52%) was related to within-individual variations, possibly indicating microbial biofilm variance at different sites within the same oral cavity. Even though both oral conditions, caries and periodontitis, are driven by oral biofilms, their aetiologies are different. Dental caries is caused by an increase in acidogenic and aciduric bacteria in the biofilm, while proteolytic bacteria and corresponding host response are involved in periodontal conditions [6, 21, 31]. However, the ecological plaque hypothesis suggests that the dynamics of biofilm, particularly its dysbiotic state, may play a more significant role in the aetiology and progression of both caries and periodontal diseases [32, 33]. A recent Canadian study showed a positive association between caries (measured as DMFS and DS) and severity of periodontitis (measured according to the classification of the Center for Disease Control and Prevention–American Academy of Periodontology) and clinical attachment loss based not only on clinical but also on microbiological measures, i.e., the association was observed between levels of *S. mutans* and periodontal pathogens such as *F. nucleatum* [8, 10]. The Cochrane systematic review concluded that flossing, in addition to toothbrushing, may reduce dental biofilm, suggesting a control of both caries and periodontitis, with the authors suggesting that interdental brushes may be more effective than floss [34]. In our study, caries was associated independently with periodontal inflammation even after the adjustment for oral biofilm control, such as tooth brushing and using interdental care products. The previously mentioned Albandar et al. longitudinal adolescent study showed that the presence of untreated carious lesions and restorations, but not inadequate oral biofilm control, can be predisposing factors for the progression of periodontal conditions [15].

According to our study, the co-occurrence of untreated carious lesions and bleeding on probing or increased pocket probing depth on the same teeth was observed in all age groups, with only a few exceptions. Additionally, the co-occurrence of restored teeth and periodontal inflammation were observed in all age groups. Our results are in line with a recent study that demonstrated confluence of different caries and periodontitis indicators across the lifespan [11]. Of note, the latter study included participants aged ≥ 13 years and discussed the coinciding onset of caries and periodontitis, which co-occur and accumulate until early adulthood, while in older age, caries grouped with increased destruction of periodontal tissues, indicated by severe clinical attachment loss (CAL) and furcation involvement. In our study, the participants were older (≥ 34 years) therefore we were not able to examine co-occurrence of caries and periodontitis indicators in younger age groups. Although the direct comparison between the two studies is not possible, it seems that the participants of the Costa and colleagues’ study had almost double the levels of decayed teeth (mean DT > 3 in 30–49 years age group in the Costa et al. study versus mean DT around 1.5 in 34–44 years group in our national study sample) [35]. Levels of PPD could not be compared as PPD was measured at site-level in Costa et al. while PPD was measured at tooth-level in the current study. Another cross-sectional study, involving a large sample of 13–20 years adolescents with very low levels of dentine caries and PPD ≥ 5 mm, examined the association between caries and periodontitis employing structural equation modelling and negative binomial regression analyses [36]. The authors found that the extent of periodontitis (number of affected sites per individual) was positively associated with the numbers of the dentine caries lesions, aligning with our findings. However, the latter study showed that the severity of periodontitis (the number of sites with CAL ≥ 3 mm and PPD ≥ 5 mm) was negatively associated with both enamel and dentine caries. The authors suggested that in deeper periodontal pockets, the amount of fermentable sugar might have been reduced, thus partially protecting the tooth surfaces from caries. The difference between the results of our and the aforementioned study may be due to the different analytical approach (individual-level versus teeth-level) and differing outcomes (caries measure in the study by Nascimento and colleagues versus periodontitis-related measures in our study), as well as differences in the levels of oral conditions and different definitions of periodontal disease. Indeed, it has been suggested that conflicting results in the literature regarding the association between caries and periodontitis may be due to the different classifications used to quantify caries and periodontitis on individual level [11].

In addition to sharing common risk factors, genetic variation regarding the susceptibility of caries and periodontal diseases may also play a role in the association between these two oral conditions. It has been proposed that the genetic variation in the salivary protein lactoferrin may reduce the formation of dental caries-related biofilm while in African American adolescents lactoferrin was also related to periodontal vulnerability [37]. Further studies exploring association between caries and periodontal diseases should include not only social and behavioural determinants, but also consider genetic variation.

The present study used a representative and relatively large sample of the adult population that had high levels of both caries and periodontitis. Additionally, the data included information about all 28 permanent teeth, rather than just a preselected index teeth. In the present study, we used data collected for the Lithuanian National Oral Health Survey 2017/2019, adhering to the WHO Basic Methods recommendations. During the clinical examination, although BOP and PPD were measured on six sites of each tooth, these were subsequently recorded at the tooth level, selecting the most severe measure for each. Therefore, not site specific but tooth level analyses were employed in our study. We have not only included proximal but also buccal and oral surfaces to represent caries status. This was important, as our previous study, which explored caries experience following the surface-based susceptibility hierarchy, showed that the highest proportion of dental caries experience was in Groups 1 and 2 of this hierarchy, which also include buccal and oral surfaces of molars [23]. Of importance, multilevel modeling was utilized, which accounted for the clustering structure of the data. Although analyses were adjusted for several common risk factors of caries and periodontal diseases, the possibility for other potential confounders cannot be ruled out.

## Conclusions

Our findings add evidence for the co-occurrence of periodontal inflammation and caries on the same teeth. This suggests the need for increased emphasis on a transdisciplinary approach in designing oral health interventions that target dental caries and periodontal disease simultaneously. In addition, longitudinal studies exploring the co-occurrence of caries and periodontal disease at the same sites, taking into consideration the levels of both conditions and genetic variation, are warranted.

### Electronic supplementary material

Below is the link to the electronic supplementary material.

Supplementary Material 1

Supplementary Material 2

## Data Availability

The data that support the findings of this study are available from the corresponding author (LS-M, linas@ofk.no) upon a reasonable request.

## Abbreviations

BOP: Bleeding on probing
CAL: Clinical attachment loss
CI: Confidence interval
DT: Decayed teeth
FT: Filled teeth
OR: Odds ratio
PPD: Pocket probing depth
WHO: The World Health Organization
