# Supplementary material for: Co-occurrence of dental caries and periodontitis: multilevel modelling approach
Source: BMC Oral Health. 2024 Jan 31;24:149. doi: 10.1186/s12903-024-03918-2 (PMC10832139; doi:10.1186/s12903-024-03918-2)
Supplement: Supplementary file 1 — Supplementary Material 1 [file 12903_2024_3918_MOESM1_ESM.docx]

**Supplementary Table 1**. Sensitivity analyses adjusted by all sociodemographic, behavioral and biological determinants in Model 2.

|  | Main effects (2) tooth-level and individual-level^^[[1]](#endnote-1)^^ | | | |  |
| --- | --- | --- | --- | --- | --- |
| ***Outcome BOP*** |  | | | |  |
| **Fixed effects** | | | **OR (95% CI)** | | |
| *No DT (ref.)* |  |  |  |  |  |
| *DT* | 1.41 (1.19-1.66) | | | |  |
| ICC | 0.2669 |  | |  |  |
| Model fit BIC | 55724.327 | | | |  |
| **Fixed effects** | | | **OR (95% CI)** | | |
| *No FT (ref.)* |  |  |  |  |  |
| *FT* | 2.08 (1.93-2.23) | | | |  |
| ICC | 0.2595 |  | |  |  |
| Model fit BIC | 87272.827 | | | |  |
| ***Outcome PPD*** | |  | | | |
| **Fixed effects** | | | **OR (95% CI)** | | |
| *No DT (ref.)* |  |  |  |  |  |
| *DT* | 1.37 (1.13-1.65) | | | |  |
| ICC | 0.4073 |  | |  |  |
| Model fit BIC | 63432.337 | | | |  |
| **Fixed effects** | | | **OR (95% CI)** | | |
| *No FT (ref.)* |  |  |  |  |  |
| *FT* | 2.02 (1.84-2.21) | | | |  |
| ICC | 0.3991 |  | |  |  |
| Model fit BIC | 97827.305 | | | |  |

1. Analyses adjusted for age, sex, education, residency, sugar-containing diet, tooth brushing frequency, use of interdental care products, last dental visit, smoking, systemic disease, use of medication and xerostomia. [↑](#endnote-ref-1)
