# Supplementary material for: Co-occurrence of dental caries and periodontitis: multilevel modelling approach
Source: BMC Oral Health. 2024 Jan 31;24:149. doi: 10.1186/s12903-024-03918-2 (PMC10832139; doi:10.1186/s12903-024-03918-2)
Supplement: Supplementary file 2 — Supplementary Material 2 [file 12903_2024_3918_MOESM2_ESM.pptx]

## Slide 1
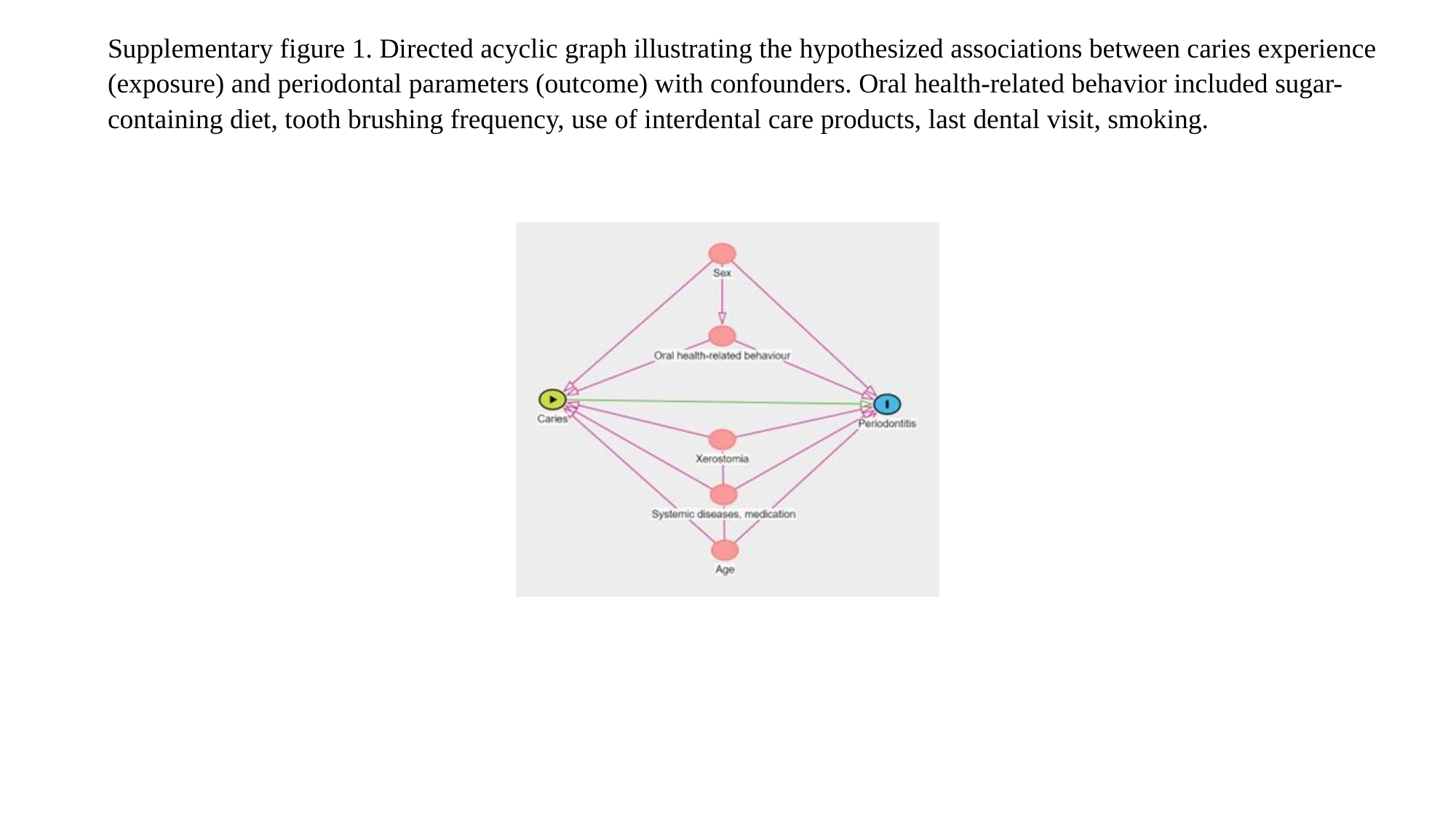

Supplementary figure 1. Directed acyclic graph illustrating the hypothesized associations between caries experience (exposure) and periodontal parameters (outcome) with confounders. Oral health-related behavior included sugar-containing diet, tooth brushing frequency, use of interdental care products, last dental visit, smoking.
